# Robust induction of neural crest cells to derive peripheral sensory neurons from human induced pluripotent stem cells

**DOI:** 10.1038/s41598-020-60036-z

**Published:** 2020-03-09

**Authors:** Yoshie Umehara, Sumika Toyama, Mitsutoshi Tominaga, Hironori Matsuda, Nobuaki Takahashi, Yayoi Kamata, François Niyonsaba, Hideoki Ogawa, Kenji Takamori

**Affiliations:** 1 Juntendo Itch Research Center (JIRC), Institute for Environmental and Gender Specific Medicine, Juntendo University Graduate School of Medicine, 2-1-1 Tomioka, Urayasu, Chiba, 279-0021 Japan; 2 Atopy (Allergy) Research Center, Juntendo University Graduate School of Medicine, 2-1-1 Hongo, Bunkyo-ku, Tokyo, 113-8421 Japan; 3 Faculty of International Liberal Arts, Juntendo University, 2-1-1 Hongo, Bunkyo-ku, Tokyo, 113-8421 Japan; 4 Department of Dermatology, Juntendo University Urayasu Hospital, 2-1-1 Tomioka, Urayasu, Chiba, 279-0021 Japan

**Keywords:** Induced pluripotent stem cells, Peripheral nervous system

## Abstract

Because intractable itch reduces quality of life, understanding the fundamental mechanisms of itch is required to develop antipruritic treatments. Itch is mediated by peripheral sensory neurons, which originate from the neural crest (NC) during development. Itch-associated signaling molecules have been detected in genetically engineered animals and in cultures of peripheral neurons from dorsal root ganglia (DRG). Ethical difficulties collecting peripheral neurons from human DRG have limited analysis of itch in humans. This study describes a method of differentiating peripheral neurons from human induced pluripotent stem cells (hiPSCs) for physiological study of itch. This method resulted in the robust induction of p75 and HNK1 double-positive NC cells from hiPSCs. The expression of NC markers *TFAP2A*, *SOX10* and *SNAI1* increased during NC induction. The induction efficiency was nearly 90%, and human peripheral neurons expressing peripherin were efficiently differentiated from hiPSC-derived NC cells. Moreover, induced peripheral neurons expressed the sensory neuronal marker BRN3A and the itch-related receptors HRH1, MRGPRX1, IL31R and IL-4R. Calcium imaging analyses indicated that these peripheral neurons included sensory neurons responsive to itch-related stimuli such as histamine, BAM8-22, IL-31 and IL-4. These findings may enable detailed analyses of human DRG neurons and may result in new therapies for intractable itch.

## Introduction

Somatic sensations, including itch and pain are mediated by primary sensory neurons, which have cell bodies in dorsal root ganglia (DRG) or trigeminal ganglia. These neurons differ in somal size, expression of ion channels and receptors, innervation territories, and electrophysiological properties^1^. Small-diameter DRG neurons with unmyelinated axons (C fibers) are the major types that mediate itch and pain^1,2^. Similar to pain, chronic and intractable itch reduces quality of life in patients with skin diseases such as atopic dermatitis (AD), which are frequently resistant to conventional treatments, such as histamine H_1_ receptor antagonists (antihistamines). Itch in atopic skin is elicited by noxious stimuli, with barrier dysfunction caused by itch-induced scratching, which are associated with the itch-scratch cycle^3^. Understanding the fundamental mechanisms of itch is required for the development of antipruritic treatments.

Studies in genetically engineered animals and rodent DRG neuron cultures have revealed signaling molecules and receptors that contribute to the induction and transmission of itch. Mas-related G protein-coupled receptors (Mrgprs), especially MrgprAs, MrgprB4, MrgprB5, MrgprC11 and MrgprD, have been reported involved in histamine-independent itch pathways in mice, with the expression of these Mrgprs restricted to small-diameter DRG neurons^4^. In humans, MRGPRXs are also selectively expressed in DRG neurons^5^. The antimalarial drug chloroquine and bovine adrenal medulla peptide 8–22 (BAM8-22) activate MrgprA3 and MrgprC11, respectively, and elicit itch in mice through a distinct signaling pathway^6^. Both chloroquine and BAM8-22 activate MRGPRX1 and elicit itch in humans^7–9^, but the downstream signaling pathway is unclear. Because the mechanisms mediating itch in humans and rodents largely differ, detailed investigations of the mechanisms mediating itch in humans therefore require human DRG neurons. For ethical reasons, however, it is difficult to collect and analyze peripheral neurons from human DRG. Therefore, this study attempted to induce peripheral sensory neurons from human induced pluripotent stem cells (hiPSCs).

During vertebrate development, peripheral sensory neurons are derived from the neural crest (NC). The NC emerges at the interface between the neural and non-neural ectoderm and differentiates into various cell types including neuron, glial cell, cranial bone, cartilage, smooth muscle and melanocyte^10^. Defects in the NC lineage are therefore responsible for various diseases, including Hirschsprung’s disease, DiGeorge syndrome, Waardenburg syndrome, Charcot-Marie-tooth disease, familial dysautonomia, congenital insensitivity to pain with anhidrosis and pediatric cancers, such as neuroblastoma^10^. To understand the pathogenesis of these NC-associated disorders, NC cells derived from human embryonic stem cells (hESCs) or hiPSCs are needed. However, most methods have assessed NC induction efficiency by measuring p75 expression alone, using a reporter gene or analyzing mRNA levels, but did not assess undifferentiated marker expression or detail the numbers of days and cells. This study describes a protocol for the highly efficient induction of NC from hiPSCs based on the NSB (Noggin and SB431542) culture system^10^. In addition, this study reports that these hiPSC-derived NC cells have the potential to differentiate into human peripheral sensory neurons that respond to itch-related stimuli.

## Results

### Specification of NC from hiPSCs

**Figure 1:**
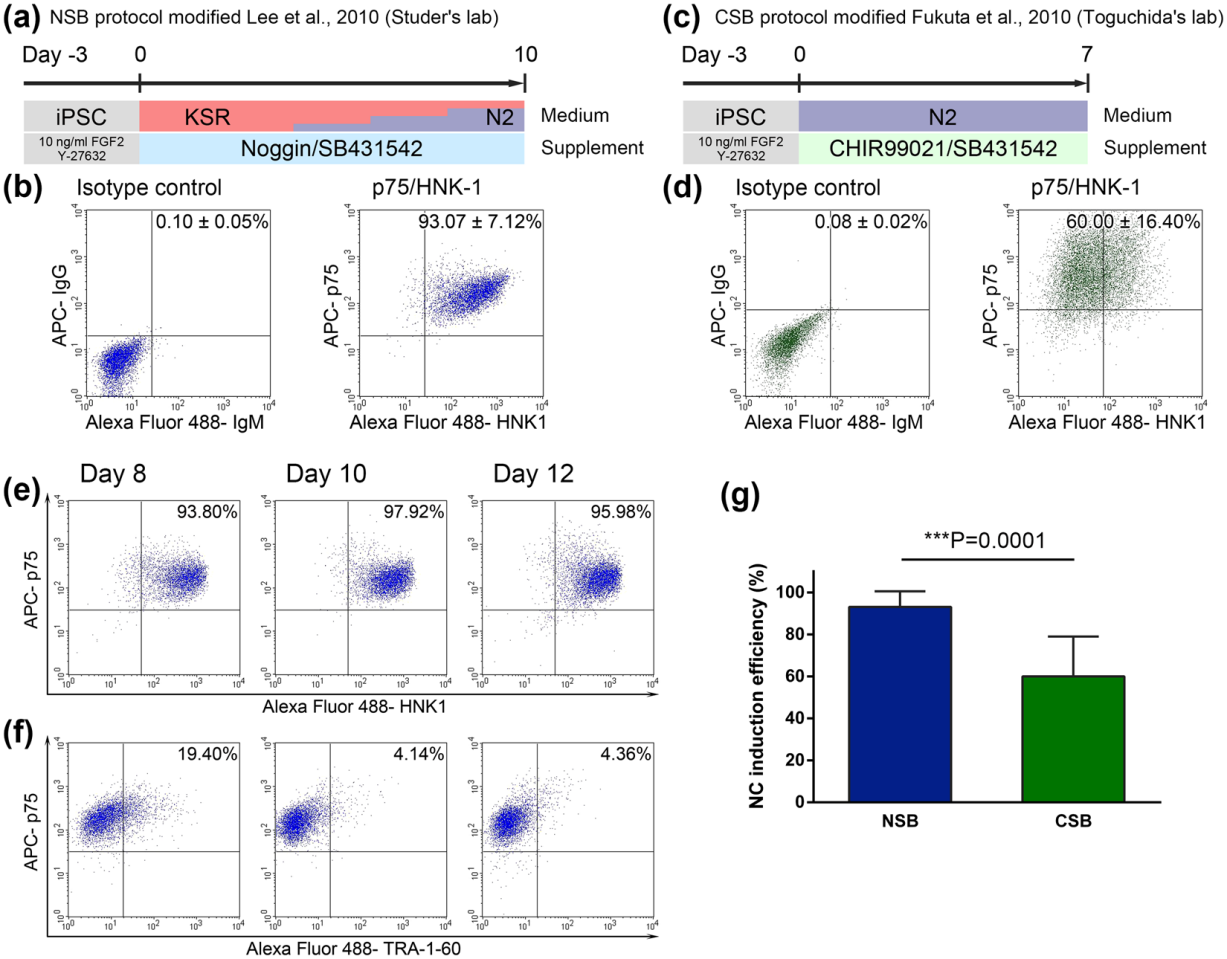
Induction of NC cells from hiPSCs. (**a**) Schematic outline of NC induction protocol with Noggin and SB431542. (**b**) Expression on day 10 of p75 and HNK1 on NC cells treated with Noggin and SB431542. (**c**) Schematic outline of NC induction protocol with CHIR99021 and SB431542. (**d**) Expression on day 7 of p75 and HNK1 on NC cells treated with CHIR99021 and SB431542. (**e**) Expression on days 8, 10 and 12 of p75 and HNK1 on NC cells induced by the NSB protocol. (**f**) Expression on days 8, 10 and 12 of p75 and TRA-1–60 on NC cells induced by the NSB protocol. Each graph shows the percentage of double-positive cells. (**g**) Comparative NC induction efficiency using the NSB (n = 13) and CSB (n = 4) protocols; results were compared statistically using Student’s t-test.

Firstly, we attempted to induce NC from hiPSCs, since NC is a progenitor cell of peripheral neuron. hiPSCs were dissociated into single cells and seeded onto Matrigel-coated dishes without feeder cells. When the cells became 50% confluent, differentiation was initiated (day 0) by replacing culture medium with differentiation medium. To determine the optimal culture condition and method, two protocols, NSB (Noggin and SB431542) and CSB (CHIR99021 and SB431542), which differed in media, inhibitors and duration of differentiation, were compared. In the NSB protocol, cells were cultured for 10 days with KnockOut Serum Replacement (KSR) or N2 supplement medium, both of which contain the SMAD signaling inhibitors Noggin and SB431542 (Fig. 1a)^10,11^. Noggin is an endogenous extracellular inhibitor that binds bone morphogenic protein (BMP), inhibiting BMP receptor activation and signaling to SMAD1/5/8. SB431542 inhibits the activation of activin receptor-like kinase (ALK) 5, which is induced by transforming growth factor (TGF)-β or activin, resulting in the suppression of SMAD2 signaling. In the CSB protocol, cells were cultured for 7 days in N2 medium containing the glycogen synthase kinase (GSK) 3β inhibitor CHIR99021 and SB431542 (Fig. 1c)^12^. The efficiency of NC induction was evaluated by fluorescence activated cell sorting (FACS) analyses of the levels of expression on cells of the NC markers p75 and HNK1, using the same clones of antibodies as used in the NSB culture system^10^. The NSB protocol increased the fraction of p75 and HNK1 double-positive cells to 93.07 ± 7.12%, indicating high efficiency NC induction (Fig. 1a,b,g). In contrast, the CSB protocol increased p75 but not HNK1 expression (Fig. 1c,d). The efficiency of NC induction using the CSB protocol was 60.00 ± 16.40% (Fig. 1d,g).

To optimize the time for differentiation using the NSB protocol, the levels of expression of both NC and hiPSC markers were analyzed on days 8, 10 and 12 of NC induction. At all three times, the fraction of double-positive cells for p75 and HNK1 was over 90% (Fig. 1e). HNK1 expression was highest on day 10 and the hiPSC marker TRA-1–60 was still expressed in about 20% of cells on day 8 (Fig. 1f). Moreover, many cells had died after 12 days (Supplementary Fig. S1). Based on these results, cells were cultured for 10 days using the NSB protocol.

To further optimize differentiation conditions, Noggin in the medium was replaced with LDN193189, which inhibits the activation of SMAD1/5/8 signaling from the BMP receptors ALK2 and ALK3 (Supplementary Fig. S2a). Increased expression of NC markers on day 10 was comparable for cells cultured with LDN193189 and Noggin (Supplementary Fig. S2b and S2e), with residual TRA-1–60 expression observed on day 8 (Supplementary Fig. S2d). These findings indicated that differentiation medium supplemented with SB431542 and Noggin or LDN193189 efficiently induced NC cells from hiPSCs. Moreover, the NSB protocol resulted in high efficiency NC induction in three other hiPSC lines, iPS-TIG120–3f7, iPS-TIG107–4f1 and iPS-TIG114–4f1 (Supplementary Fig. S2f).

### Induction of NC from hiPSCs by Noggin and SB431542

**Figure 2:**
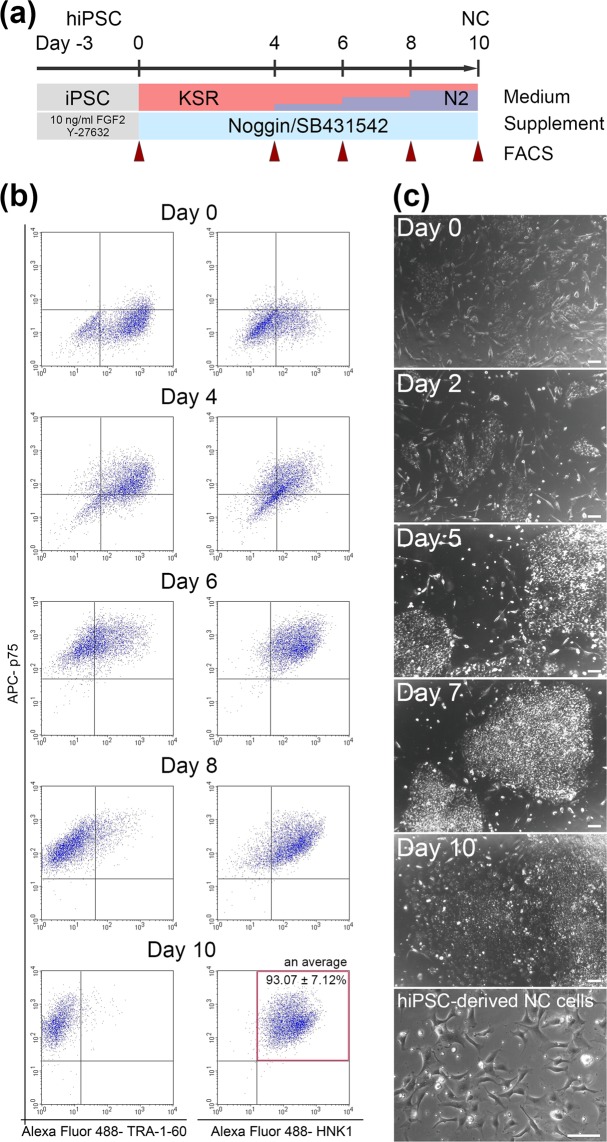
Expression of p75 and HNK1 during NC induction. (**a**) Schematic overview of the established NC induction protocol using Noggin and SB431542. The cells were analyzed by FACS on days 0, 4, 6, 8 and 10, at the time medium was changed (arrowheads). (**b**) Flow cytometry analyses of p75 and TRA-1–60 (left) or HNK1 (right) expression during NC induction. (**c**) Cell morphology during and after (lowest) NC induction. Scale bars, 100 µm.

Differentiation in medium supplemented with Noggin and SB431542 (Fig. 2a) resulted in the effective generation of NC cells from hiPSCs, with an average NC induction efficiency of 93.07 ± 7.12% (n = 13). FACS analyses showed increasing p75 expression until day 6, whereas HNK1 expression increased and TRA-1–60 expression decreased through day 10 (Fig. 2b). During NC induction, these cells gradually proliferated and changed morphologically (Fig. 2c).

**Figure 3:**
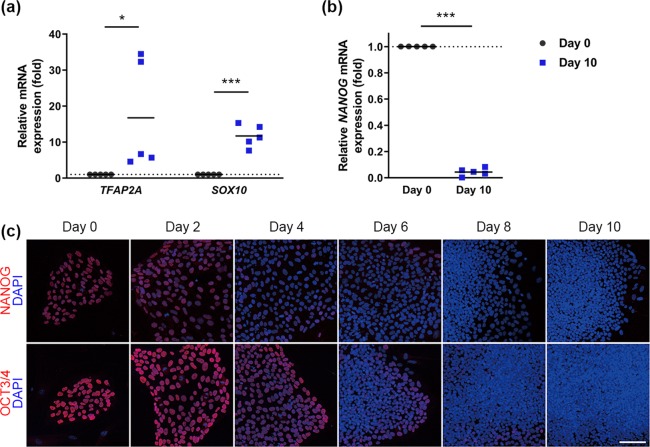
Progression of differentiation from hiPSCs to NC lineage. (**a**,**b**) Expression of *TFAP2A* and *SOX10* (**a**) and *Nanog* (**b**) mRNAs by quantitative real-time RT-PCR, normalized relative to the expression of *RPS18* mRNA in the same samples. Values represent fold changes in gene expression compared with Day 0 and the horizontal lines represent the mean of five independent experiments. *P < 0.05, ***P < 0.0001 compared with the same gene on Day 0 by Student’s t-test. (**c**) Immunocytochemistry showing reduced expression of the pluripotent cell markers NANOG and OCT3/4 (red) on the indicated days. Scale bar, 100 µm.

The expression of the NC markers *TFAP2A*, *SOX10*, *SNAI1*, *SNAI2* and *SOX9* increased during the induction of NC for 10 days (Fig. 3a, Supplementary Fig. S3a and S3c), whereas the expression of hiPSC markers, such as NANOG and OCT3/4, gradually decreased (Fig. 3b,c). The central nervous system (CNS) marker PAX6 was detected in a few cells and the expression of *PAX6* mRNA was much lower than that in human brain as a positive control (Supplementary Fig. S3b and S3d).

### Expression patterns of NC markers in hiPSC-derived NC cells

**Figure 4:**
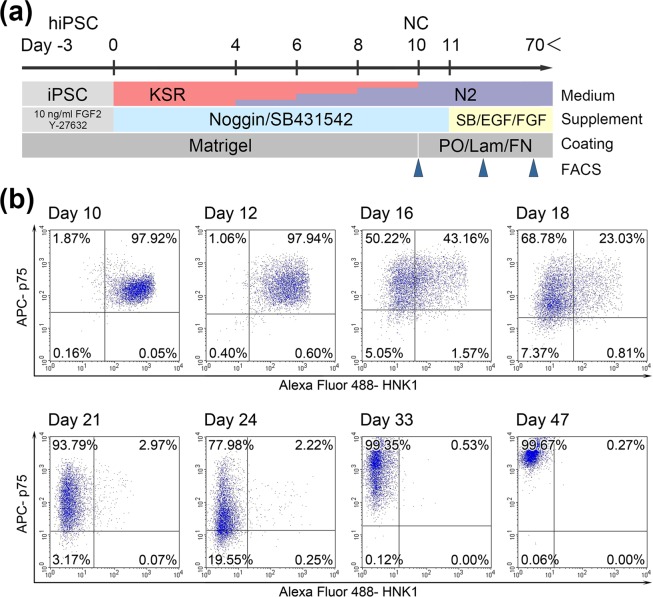
Alterations in p75 and HNK1 expression on hiPSC-derived NC cells. (**a**) Schematic overview of the established NC induction and subsequent culture protocols. Medium was changed on days −2, 0, 2, 4, 6 and 8. On day 10, hiPSC-derived NC cells were passaged onto PO/Lam/FN-coated plates (arrowheads), followed by suitable passage and analysis. (**b**) Flow cytometry analyses of p75 and HNK1 expression on hiPSC-derived NC cells at the indicated times.

Because p75 and HNK1 were expressed on almost all hiPSC-derived NC cells, these cells were seeded onto poly-L-ornithine/laminin/fibronectin (PO/Lam/FN)-coated dishes without sorting after NC induction for 10 days, and incubated with N2 medium containing SB431542, 10  ng/ml EGF and 10  ng/ml FGF2 (Fig. 4a). Flow cytometry analyses of these hiPSC-derived NC cells showed that the levels of HNK1 expression were not sustained, with almost all HNK1-expressing cells disappearing by day 24 (i.e. about 2 weeks after NC induction) (Fig. 4b). In contrast, the levels of p75 expression were only slightly decreased in hiPSC-derived NC cells, with these cells subsequently showing robust p75 expression at day 33 (i.e. about 3 weeks after NC induction) and even higher p75 expression at day 47 (Fig. 4b). The levels of p75 and HNK1 expression in hiPSC-derived NC cells incubated with N2 medium containing SB431542, 20  ng/ml EGF and 20  ng/ml FGF2 were comparable with those in cells incubated with N2 medium containing SB431542, 10  ng/ml EGF and 10  ng/ml FGF2 (data not shown).

### Differentiation of peripheral neurons from hiPSC-derived NC cells

**Figure 5:**
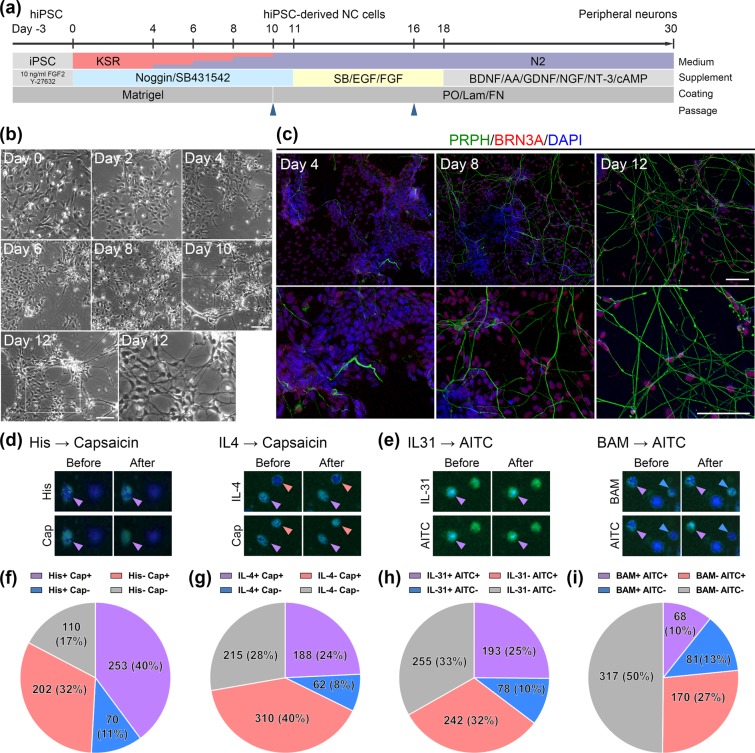
Peripheral sensory neurons from hiPSC-derived NC cells. (**a**) Schematic outline of the peripheral neuronal differentiation protocol following NC induction. (**b**) Cell morphology during neuronal differentiation. Day 0 corresponds to Day 18 in (**a**). (**c**) Immunocytochemistry of PRPH (green) and BRN3A (red) expression, showing the differentiation and growth of peripheral sensory neurons. Day 4 corresponds to Day 22 in (**a**). (**d**,**e**) Fluorescence microscopic image of calcium response following application of Histamine or IL-4 and capsaicin (**d**) and application of IL-31 or BAM8-22 and AITC (**e**). The arrowhead colors reflect colors labeled by responsiveness in the pie chart below. (**f**–**i**) Proportion of induced peripheral neurons responsive to various stimuli. Each pie chart shows the percentages of the cells that responded to either or both itch-related stimuli and agonists in a given experimental sequence: (**f**) Histamine followed by capsaicin (n = 635); (**g**) IL-4 followed by capsaicin (n = 775); (**h**) IL-31 followed by AITC (n = 768); (**i**) BAM8–22 followed by AITC (n = 636). +, positive response; −, no response. Scale bars, 100 µm. His, histamine; Cap, capsaicin; BAM, BAM8-22.

To induce peripheral neurons, hiPSC-derived NC cells were replated on PO/Lam/FN-coated dishes and incubated with N2 medium containing brain-derived neurotrophic factor (BDNF), ascorbic acid (AA), glial cell line-derived neurotrophic factor (GDNF), nerve growth factor (NGF), neurotrophin-3 (NT-3) and cyclic adenosine monophosphate (cAMP) (Fig. 5a). Morphological changes were initiated after 6 days, with differentiation into neuron-like cells observed after culture of hiPSC-derived NC cells for 12 days (Fig. 5b). These neuron-like cells expressed brain-specific homeobox/POU domain protein (BRN) 3 A and peripherin (PRPH), which are markers of sensory and peripheral neurons, respectively (Fig. 5c). Some of these cells were nociceptive neurons expressing histamine H_1_ receptor (HRH1), transient receptor potential cation channel V1 (TRPV1) or A1 (TRPA1) with PRPH (Supplementary Fig. S4a), and expressed higher levels of *HRH1*, *TRPV1* and *TRPA1* mRNAs than hiPSC-derived NC cells (Supplementary Fig. S4d). The expression levels of *BRN3A*, gastrin-releasing peptide (*GRP*) and interleukin (IL)−4 receptor (*IL4R)* mRNAs in these neuron-like cells were comparable with those in human DRGs (hDRG) (Supplementary Fig. S4b and S4c). Moreover, these cells expressed higher levels of *MRGPRX1*, *IL31RA*, ISL LIM homeobox 1 (*ISL1)* and tubulin beta 3 class III (*TUBB3)* mRNAs than hiPSC-derived NC cells (Supplementary Fig. S4b and S4c). Many peripheral neurons had differentiated from hiPSC-derived NC cells 2 weeks after NC induction (Supplementary Fig. S5a), with fewer differentiating after 3 weeks (Supplementary Fig. S5b). Moreover, many more peripheral neurons had differentiated from NC cells induced by Noggin than by LDN193189 (Supplementary Fig. S5c and S5d).

### Functional analyses of peripheral neurons from hiPSC-derived NC cells

The response of peripheral neurons from hiPSC-derived NC cells to chemical stimuli related to itch was analyzed by calcium imaging. Since these neurons expressed IL-31RA, IL-4R, MRGPRX1, HRH1, TRPV1 and TRPA1, the responsiveness to IL-31, IL-4, BAM8-22, histamine, TRPV1 agonist capsaicin and TRPA1 agonist allyl isothiocyanate (AITC) was imaged. After the initial application of a given agent, another agent was applied to peripheral neurons from hiPSC-derived NC cells 3  min later to test for self- or cross-sensitization. Representative images of calcium influx in induced peripheral neurons are shown in Figs. 5d,e. One cell responded to both histamine and capsaicin (arrowhead in Fig. 5d), and another cell responded to capsaicin but not histamine (Fig. 5d). Similarly, one cell responded to IL-31 and AITC (arrowhead in Fig. 5e and Supplementary Fig. S4f). Of these cells, 50.9% were responsive to histamine (Fig. 5f), 32.3% to IL-4 (Fig. 5g), 35.3% to IL-31 (Fig. 5h), and 23.4% to BAM8-22 (Fig. 5i). These peripheral neurons also responded to capsaicin and AITC (Fig. 5f–i).

## Discussion

The present study describes a highly efficient method of inducing NC cells from hiPSCs and shows that these NC cells could be differentiated into peripheral sensory neurons. In most previous studies, the efficiency of NC induction from hiPSCs was assessed only by analyzing p75 expression, using a reporter gene or mRNA expression alone. In addition, these studies did not precisely compare duration of culture or cell number. The first NSB culture system method reported an NC induction efficiency of about 10%^10^. Therefore, the duration of differentiation and cell number for efficient induction of the NC lineage were investigated by analyzing the expression of hiPSC and NC markers and cell viability. Our protocol, using a modified NSB culture system, showed that the increase in HNK1 expression lagged behind the increase in p75 expression, with a rate approximately equal to decreases in TRA-1–60 and OCT3/4 expression. Induction at 50% confluence was suitable for survival, since a lower density of cells at initial induction reduced the total number of hiPSC-derived NC cells, whereas a higher density increased the percentage of dead cells. Thus, our protocol resulted in the robust induction of hiPSC-derived NC cells with an efficiency of nearly 90% after just 10 days.

Using the CSB protocol with a GSK3β inhibitor also showed that differentiation medium containing CHIR99021 and SB431542 increased p75 expression to levels consistent with those of previous studies^12^, whereas HNK1 expression did not increase. This finding indicated that the protocol using a GSK3β inhibitor had a low efficiency of NC induction. In contrast, differentiation medium containing LDN193189 and SB431542 resulted in a high efficiency of NC induction, comparable to that of medium containing Noggin and SB431542. Notably, hiPSC-derived NC cells induced with LDN193189 differentiated into fewer peripheral neurons, suggesting that Noggin and LDN193189 have distinct effects on NC induction. Besides ALK2 and ALK3, which are inhibited by LDN193189, BMPs bind ALK6, suggesting that ALK6 is important for peripheral neuronal differentiation of hiPSC-derived NC cells.

A major alteration in p75 and HNK1 expression by hiPSC-derived NC cells was observed nearly a month after NC induction. Two weeks after NC induction, these cells no longer expressed HNK1, although p75 expression was sustained. This finding had not been reported in studies of gene expression profiles in induced NC cells, because HNK1 is carbohydrate chain and detectable only by immunostaining^12,13^. Moreover, global gene expression profiles were different from original pluripotent stem cells or NC induction protocols and not completely invariable through long-term culture of hiPSC-derived NC cells^12^. Long-term culture of hiPSC-derived NC cells decreased the differentiation efficiency of peripheral neurons, indicating that hiPSC-derived NC cells cultured for approximately 2 weeks lose the capacity to differentiate into peripheral neurons. These results indicate that gene expressions, protein modifications and potency of differentiation change gradually during culture of hiPSC-derived NC cells. Stable induction of human peripheral sensory neuron will require studies to investigate the culture conditions of hiPSC-derived NC cells.

NC cells induced by our NSB protocol were differentiated to peripheral sensory neurons expressing the sensory neuron marker BRN3A and the peripheral neuron marker PRPH. These peripheral neurons expressed lower levels of the peripheral motor neuron marker *ISL1* than of *BRN3A*. GRP is a neuropeptide mediating histamine-independent itch in peripheral neurons^14^, with the expression levels of GRP being comparable in induced peripheral neurons and hDRGs. Thus, these findings suggest that peripheral sensory neurons were efficiently induced by our method.

Some of these induced peripheral neurons also expressed HRH1, MRGPRX1, IL31R, IL4R, TRPV1 and TRPA1. These molecules are expressed on human nociceptive neurons and respond to histamine, BAM8-22, IL-31, IL-4, capsaicin, and AITC, respectively^4,15,16^. Functional analyses by calcium imaging indicated that induced peripheral neurons were responsive to these itch-related stimuli. Stimulation with histamine, the most known pruritogen, evokes calcium influx via TRPV1 channels and the excitation of sensory neurons. In actuality, 40% of induced peripheral neurons responded to histamine and the TRPV1 agonist capsaicin. We found that 11% of peripheral neurons responded to histamine but not capsaicin, suggesting that calcium influx was evoked independently of TRPV1 in these cells. BAM8-22 evokes histamine-independent itch through binding to MRGPRX1 in humans, but the TRP channel associated with MRGPRX1-mediated itch remains unclear. In mice, TRPA1 has been reported involved in BAM8-22-evoked itch^6^. Calcium imaging showed that 10% of induced peripheral neurons responded to BAM8-22 and the TRPA1 agonist AITC. We found that 13% of cells responded to BAM8-22 but not AITC, suggesting that calcium influx was evoked independently of TRPA1 in these neurons. Peripheral neurons responding to capsaicin but not histamine (32%) or to AITC but not BAM8-22 (27%) may be involved in mediating pain. These findings suggest that induced peripheral neurons include the sensory neurons involved in the transmission of histamine-dependent itch, histamine-independent itch and pain.

Recently, IL-31 and IL-4 have been implicated in intractable itch associated with atopic dermatitis. IL-31 directly stimulates mouse sensory neurons expressing IL-31R via TRPV1 or TRPA1^17^. In the present study, 10% of induced peripheral neurons responded to IL-31 but not AITC, suggesting that calcium influx was evoked by TRPV1 in these cells. Both IL-31 and AITC activate 25% of induced peripheral neurons, which may be excited by TRPA1. The Th2 cytokine IL-4 activates human and mouse sensory neurons, exaggerating itch sensation induced by histamine or IL-31^16^. The present study showed that 32.3% of induced peripheral neurons responded to IL-4, including 24.3% that also responded to capsaicin. These results suggest that peripheral neurons from hiPSC-derived NC cells react with various stimuli associated with itch and may therefore be useful in studies investigating the fundamental mechanisms of itch elicitation in human DRG neurons.

In conclusion, this study described a method for the robust induction of NC from hiPSCs, and for differentiating these NC cells into human peripheral sensory neurons responding to itch-related stimuli. These methods and findings may enable analyses of human sensory neurons associated with itch and may be useful in the development of new therapies for chronic and intractable itch.

## Methods

### hiPSC culture

The hiPSC line 201B7 was provided by the RIKEN BRC through the National Bio-Resource Project of the MEXT, Japan. The hiPSC lines, iPS-TIG120–3f7 iPS-TIG107–4f1 and iPS-TIG114–4f1, were provided by the Center for iPS Cell Research and Application, Kyoto University through the JCRB Cell Bank (National Institutes of Biomedical Innovation, Health and Nutrition, Japan). hiPSCs were grown on mitomycin C-treated mouse embryonic fibroblasts (MEFs; ReproCELL). MEFs were cultured in Dulbecco’s modified Eagle’s medium (DMEM; Sigma-Aldrich) supplemented with 10% fetal bovine serum (FBS; Japan Bio Serum) on cell culture dishes coated with 0.1% gelatin (Sigma-Aldrich). hiPSCs were cultured with DMEM/F12 (Gibco) containing 20% KnockOut Serum Replacement (KSR; Invitrogen), 1% L-Glutamine (Invitrogen), 1 × penicillin/streptomycin (Sigma-Aldrich), 1 × MEM non-essential amino acids (MEM-NEAA; Sigma-Aldrich), 0.1  mM 2-mercaptoethanol (ME; Wako Pure Chemical Industries) and 5  ng/ml recombinant human fibroblast growth factor 2 (rhFGF2; Chemicon), with the medium changed every day. hiPSCs were passaged every week using a dissociation solution (CTK solution; ReproCELL), with a split ratio of 1:3.

### hiPSC differentiation to NC lineage

hiPSC colonies were dissociated into single cell by pipetting and passage through a 40 μm cell strainer (BD Biosciences). hiPSCs, at a density of 1.1–1.2 × 10^4^ cells per cm^2^ in hiPSC medium containing 10  μM Y-27632 (ROCK inhibitor; Wako Pure Chemical Industries), were plated onto cell culture dishes coated with growth factor-reduced Matrigel (BD Biosciences). The medium was replaced the next day with hiPSC medium containing 10  ng/ml rhFGF2 and 10  μM Y-27632. When the cells became 50% confluent, differentiation was initiated (day 0) by replacing the medium with KSR medium, consisting of KnockOut DMEM (Invitrogen), 15% KSR, 1% L-Glutamine, 1 × penicillin/streptomycin, 1 × MEM-NEAA, and 0.1  mM 2-ME, supplemented with 500  ng/ml Noggin (R&D Systems) and 10  μM SB431542 (Stemgent Inc.). After 48  hours (day 2), the medium was replaced with the same medium, followed by slow switching from KSR to N2 on days 4 (75% KSR, 25% N2), 6 (50% KSR, 50% N2), 8 (25% KSR, 75% N2), and 10 (100% N2) (see Fig. 3a). N2 medium consisted of DMEM/F12, 0.2% Insulin solution (Sigma-Aldrich) and 1 × N2 supplement with Transferrin (Apo) (Wako Pure Chemical Industries). In some experiments, Noggin was replaced by 1  μM CHIR99021 (Wako Pure Chemical Industries) and 500  nM LDN193189 (Sigma-Aldrich).

hiPSC-derived NC cells were cultured in N2 medium supplemented with 10  µM SB431542, 10  ng/ml epidermal growth factor (EGF; Sigma-Aldrich) and 10  ng/ml hrFGF2 on plates coated with poly-L-ornithine/laminin/fibronectin (PO/Lam/FN), consisting of 15  μg/ml poly-L-ornithine (Sigma-Aldrich), 1  μg/ml ultra-pure laminin (BD Biosciences) and 10  μg/ml human plasma fibronectin (Millipore), with medium changed every 2 or 3 days. The first passage, on day 10 of NC induction, was performed using Accutase (Innovative Cell Technologies), with a split ratio of 1:2. Subsequent passages every 4–6 days were performed using Accutase with a split ratio of 1:4–1:6.

### Peripheral neuronal differentiation of hiPSC-derived NC cells

Human peripheral neurons were induced as described^10^. Briefly, NC cells were plated at a density of 1 × 10^5^ cells per cm^2^ onto cell culture dishes coated with PO/Lam/FN. After 48  hours, the medium was replaced with N2 medium containing 10  ng/ml brain-derived neurotrophic factor (BDNF; Sigma-Aldrich), 200  μM ascorbic acid (AA; Sigma-Aldrich), 10  ng/ml glial cell line-derived neurotrophic factor (GDNF; Sigma-Aldrich), 10  ng/ml nerve growth factor (NGF; R&D Systems), 10  ng/ml neurotrophin-3 (NT-3; R&D Systems) and 0.5  mM cyclic adenosine monophosphate (cAMP; Tokyo Chemical Industry). The medium was changed every 3 or 4 days, with the cells cultured for 12–14 days for peripheral neuronal differentiation.

### Antibodies

The primary antibodies used in this study were mouse anti-CD57/HNK-1 antibody (500  ng/10^6^ cells; Sigma-Aldrich), mouse isotype control antibody (500  ng/10^6^ cells; Sigma-Aldrich), APC-conjugated mouse anti-p75 (CD271) antibody (250  ng/10^6^ cells; BioLegend), APC-conjugated mouse isotype control antibody (250  ng/10^6^ cells; BioLegend), Alexa Fluor® 488-conjugated mouse anti-TRA-1–60 antibody (750  ng/10^6^ cells; BioLegend), Alexa Fluor® 488-conjugated mouse isotype control antibody (750  ng/10^6^ cells; BioLegend), rabbit anti-NANOG antibody (1:500 dilution; System Biosciences), rabbit anti-OCT3/4 antibody (1:500 dilution; System Biosciences), mouse anti-SOX10 antibody (1:500 dilution; Santa Cruz), mouse anti-PAX6 antibody (1:500 dilution; Santa Cruz), mouse anti-Peripherin antibody (1:1,000 dilution; Santa Cruz), goat anti-Peripherin antibody (1:1,000 dilution; Santa Cruz), rabbit anti-Brn3a antibody (1:600 dilution; Chemicon), goat anti-HRH1 antibody (1:50 dilution; Novus Biologicals), guinea pig anti-TRPV1 antibody (1:100 dilution; Neuromics) and rabbit anti-TRPA1 antibody (1:500 dilution; GeneTex). Secondary antibodies conjugated to Alexa Fluor dye were purchased from Molecular Probes and diluted 1:1,000.

### Flow cytometry

Cells were harvested with Accutase and stained for 20  minutes at 4 °C with primary antibodies against HNK1. The cells were washed once with FACS buffer (phosphate-buffered saline containing 2% FBS) and incubated with the appropriate secondary antibody or fluorescent protein conjugated antibody for 20  minutes at 4 °C in the dark. The cells were washed once with FACS buffer and analyzed on a FACS Caliber (BD Biosciences). Data were analyzed with CellQuest pro software (BD Biosciences).

### Immunocytochemistry

Cultured cells were fixed with 4% paraformaldehyde (Nacalai Tesque) for 10  minutes, washed with phosphate-buffered saline, and permeabilized with 0.1% Triton X-100 (Sigma-Aldrich) in phosphate-buffered saline for 15  minutes. Following incubation for 1  hour at room temperature with primary antibodies against NANOG, OCT3/4, SOX10, HRH1, TRPV1 and TRPA1, the cells were incubated with Alexa Fluor dye-conjugated secondary antibodies for 30  minutes at room temperature and mounted in Vectashield mounting medium (Vector Laboratories). Antibodies were diluted with Signal Enhancer HIKARI (Nacalai Tesque). The samples were counterstained with 4′,6-diamidino-2-phenylindole (DAPI; Wako Pure Chemical Industries). Images were acquired using a confocal laser-scanning microscope (DMIRE2; Leica Microsystems).

### Preparation of total RNA and quantitative real-time RT-PCR analysis

Total RNA was extracted from cultured cells using RNeasy Mini Kits (Qiagen), according to the manufacturer’s instructions. Human Dorsal Root Ganglion Total RNA and Human Brain Total RNA were purchased from Clontech. Samples were reverse transcribed (RT) with PrimeScript RT reagent kit (TaKaRa), according to the manufacturer’s protocol. Quantitative real-time PCR was performed on a Fast Real-Time PCR system (Applied Biosystems) using a SYBR Premix Ex Taq (TaKaRa) and the primers listed in Supplementary Table 1. Each sample was analyzed in triplicate; the measured mRNA levels in each sample were normalized to that of mRNA encoding an internal reference, ribosomal protein S18 (RPS18); and the level of expression of individual genes was reported relative to its expression in control cells before induction.

### Calcium imaging

Peripheral neurons from hiPSC-derived NC cells were loaded with 10  μg/ml Fura2-AM (Dojindo Laboratories) and 0.04% Pluronic F-127 (Dojindo Laboratories) by incubation at 37 °C for 1  hour, followed by destaining for 30  minutes in Ringer’s solution (pH7.4; 140  mM NaCl, 4  mM KCl, 10 mM N-2-hydroxyethylpiperazine-N′−2-ethanesulfonic acid (HEPES), NaH_2_PO_4_, 2  mM CaCl_2_, 1  mM MgCl_2_, 4.54  mM NaOH). Imaging was performed in Ringer’s solution at a constant flow rate of 250  ml/h. Fluorescent images were obtained with a 20 × objective (aperture 1.3) using a LEICA DMIRE2 inverted epifluorescence microscope with an Electron-multiplying CCD camera (Cool SNAP; Sutter Instrument), equipped with standard 340- and 380-nm filters controlled by a Ludl Mac6000 shutter using MetaFluor software (Nihon Molecular Devices). Exposure times were 300–600  ms and images were taken every 3  seconds. Baseline imaging was recorded for 30  seconds before the addition of the agonists. Two of the following agents were delivered in randomized order: 300  μM chloroquine (Sigma-Aldrich), 10  μM BAM8-22 (Abcam), 100  μM histamine (Sigma-Aldrich), 200  μM AITC (mustard oil; Sigma-Aldrich), 1  μM capsaicin (Sigma-Aldrich), 300  nM IL-4 (BioLegend) and 300  nM IL-31 (PeproTech). The duration of each stimulus was 30  seconds. Ratios were normalized to baseline. Cells were judged to be sensitive to an applied agent if the ratio was >10% higher than the resting level^18^. Only cells responsive to high-K+ were included for analysis. Data were analyzed with MetaFluor software (Nihon Molecular Devices).

### Statistical analysis

Data were analyzed using Prism 6 (GraphPad Software Inc.). In all analyses, P < 0.05 was considered statistically significant.

## Supplementary information

Supplementary Information.
