# Supplementary material for: Robust induction of neural crest cells to derive peripheral sensory neurons from human induced pluripotent stem cells
Source: Sci Rep. 2020 Mar 9;10:4360. doi: 10.1038/s41598-020-60036-z (PMC7063040; doi:10.1038/s41598-020-60036-z)
Supplement: Supplementary file 1 — Supplementary Information. [file 41598_2020_60036_MOESM1_ESM.pdf]

Supplementary Information

Robust induction of neural crest cells to derive peripheral sensory neurons from human induced pluripotent stem cells

Yoshie Umehara[1,2], Sumika Toyama[1], Mitsutoshi Tominaga[1], Hironori Matsuda[1], Nobuaki Takahashi[1], Yayoi Kamata[1], François Niyonsaba[2,3], Hideoki Ogawa[1], Kenji Takamori[1,2*]

[1]Juntendo Itch Research Center (JIRC), Institute for Environmental and Gender Specific Medicine, Juntendo University Graduate School of Medicine, 2-1-1 Tomioka, Urayasu, Chiba 279-0021, Japan

[2]Atopy (Allergy) Research Center, Juntendo University Graduate School of Medicine, 2-1-1 Hongo, Bunkyo-ku, Tokyo 113-8421, Japan

[3]Faculty of International Liberal Arts, Juntendo University, 2-1-1 Hongo, Bunkyo-ku, Tokyo 113-8421, Japan

[4]Department of Dermatology, Juntendo University Urayasu Hospital, 2-1-1 Tomioka, Urayasu, Chiba 279-0021, Japan

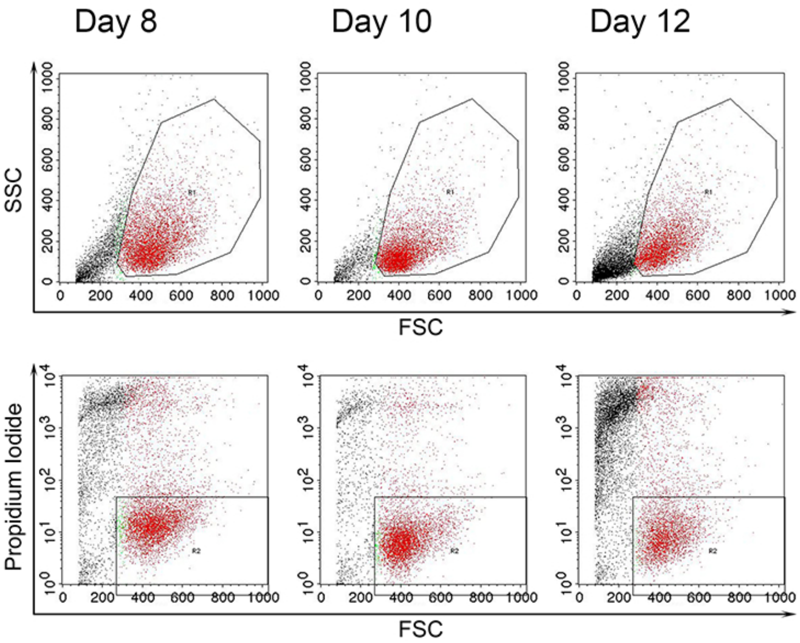

Supplementary Figure S1. State of hiPSC-derived NC cells using the NSB protocol
SSC (side scatter) and FSC (forward scatter) FACS plots (top) and propidium iodide staining (bottom) of 201B7-derived NC cells generated by the NSB protocol on days 8, 10 and 12, corresponding to Fig. 1e and 1f.

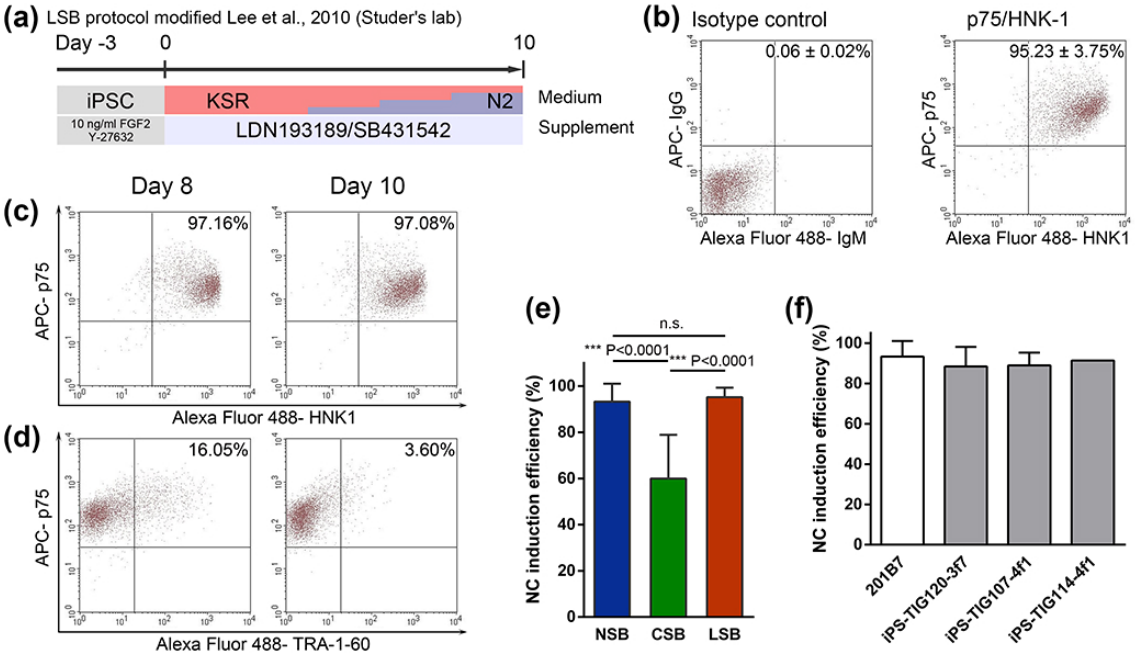

Supplementary Figure S2. Induction of NC cells by LDN193189 in place of Noggin

(a) Schematic outline of the NC differentiation protocol with LDN193189 and SB431542. (b) Expression on day 10 of p75 and HNK1 on NC cells induced from 201B7 by LDN193189 and SB431542. (c and d) Expression on days 8 and 10 of p75 and HNK1 (c) or TRA-1-60 (d) on NC cells induced by LDN193189 and SB431542. Each graph shows the percentage of double-positive cells. (e) Comparative efficiency of NC induction from 201B7 cells using the NSB (n=13), CSB (n=4) and LSB (n=6) protocols; results were compared statistically by one-way ANOVA with Tukey's multiple comparison tests. (f) Efficiency of NC induction from 201B7 (n=13), iPS-TIG120-3f7 (n=4), iPS-TIG107-4f1 (n=3) and iPS-TIG114-4f1 (n=1) cells using the NSB protocol. One-way ANOVA with Tukey's multiple comparison tests showed no significant differences, except for iPS-TIG114-4f1 cells.

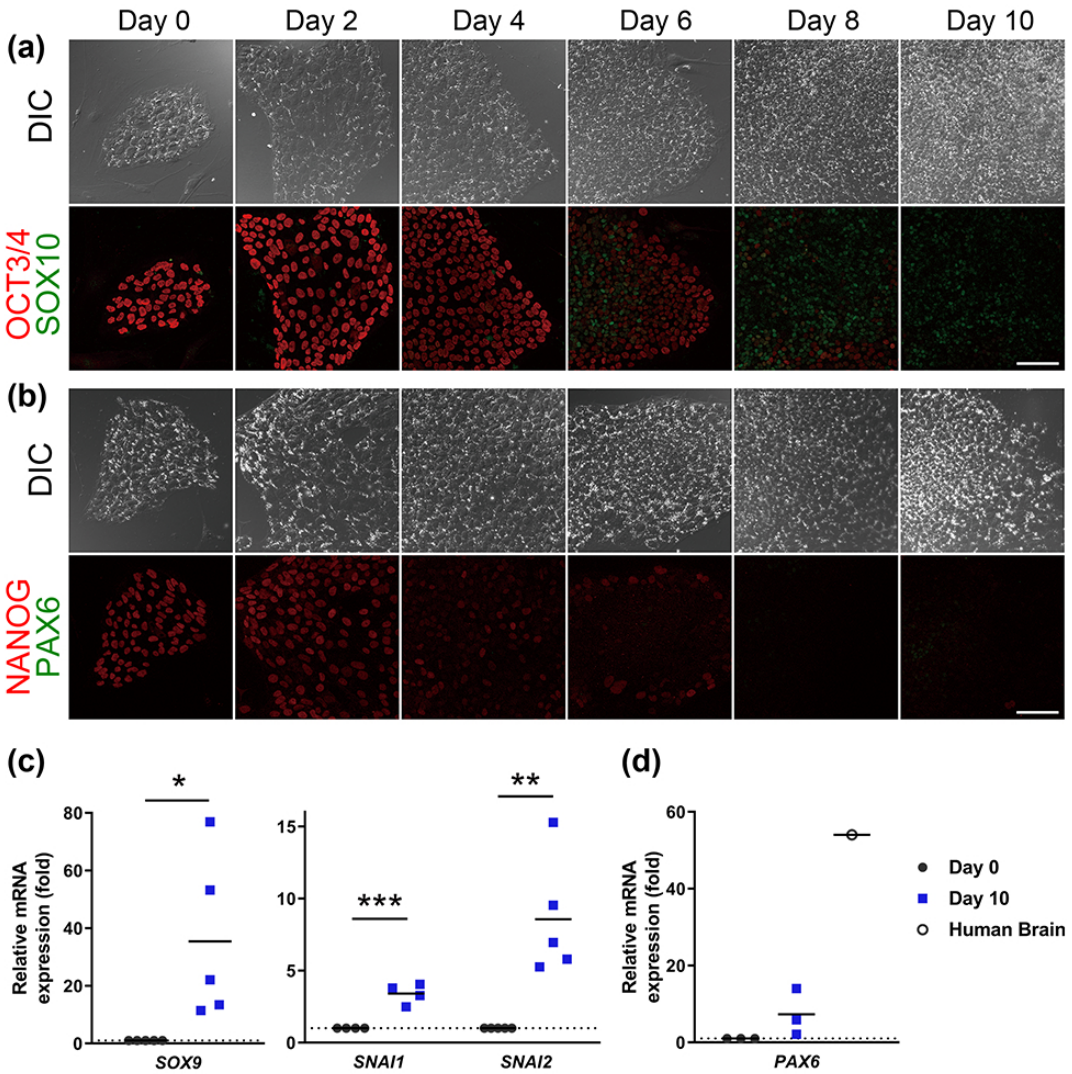

Supplementary Figure S3. Expression of hiPSC and NC markers during NC induction

(a) Immunocytochemistry showing the expression of the hiPSC marker OCT3/4 (red) and the NC marker SOX10 (green) and differential interference contrast (DIC) microscopic analysis during NC induction. Scale bar, 100 µm. (b) Immunocytochemistry showing the expression of the hiPSC marker NANOG (red) and the CNS marker PAX6 (green) and DIC microscopic analysis during NC induction. Scale bars, 100 µm. (c and d) Expression of *SOX9*, *SNAI1*, *SNAI2*, and *PAX6* mRNAs by quantitative real-time RT-PCR, normalized relative to the expression of RPS18 mRNA in the same samples. Values represent fold changes in gene expression compared with Day 0 and represent the mean ± SD of five independent experiments. * $P<0.05$, ** $P<0.001$, *** $P<0.0001$ compared with the same gene on Day 0 by Student's t-test.

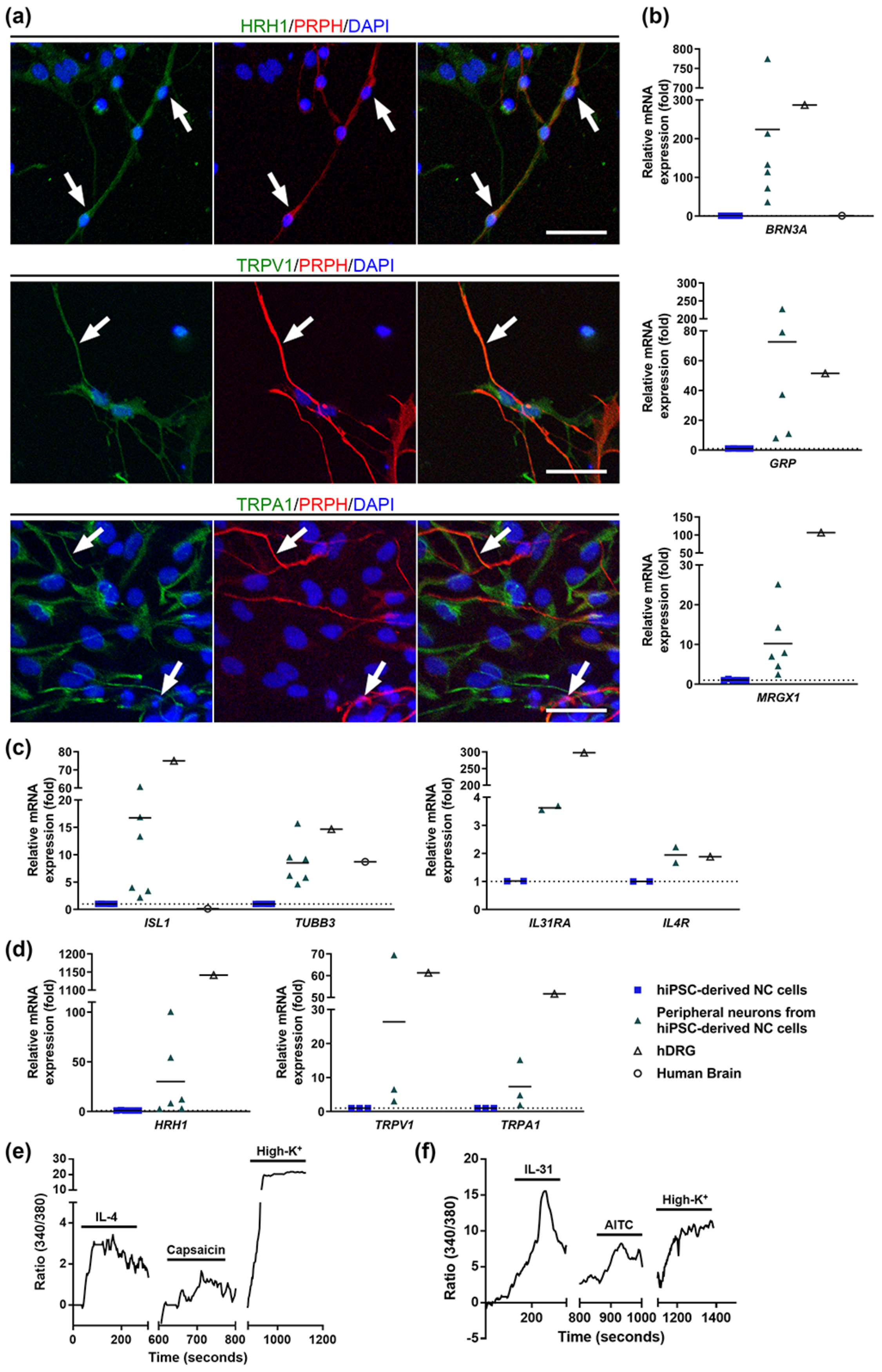

(a)
HRH1/PRPH/DAPI
TRPV1/PRPH/DAPI
TRPA1/PRPH/DAPI
(b)
Relative mRNA expression (fold)
BRN3A
GRP
MRGX1
(c)
ISL1
TUBB3
IL31RA
IL4R
(d)
HRH1
TRPV1
TRPA1
hiPSC-derived NC cells
Peripheral neurons from hiPSC-derived NC cells
hDRG
Human Brain
(e)
Ratio (340/380)
IL-4
Capsaicin
High-K+
Time (seconds)
(f)
IL-31
AITC
High-K+
Time (seconds)

Supplementary Figure S4. Expression of sensory neuronal markers by differentiated peripheral neurons

(a) Immunocytochemistry showing the expression of HRH1, TRPV1 or TRPA1 with PRPH on peripheral neurons from hiPS-derived NC cells. Scale bars, 50 μm.

(b–d) Expression of *BRN3A*, *ISL1*, *TUBB3*, *MRGPRX1*, *GRP*, *IL31R*, *IL4R*, *HRH1*, *TRPV1* and *TRPA1* mRNAs in peripheral neurons differentiated from hiPSC-derived NC cells by quantitative real-time RT-PCR, normalized relative to the expression of RPS18 mRNA. Values represent the mean ± SD fold changes in gene expression compared with hiPSC-derived NC cells.

(e and f) Representative graph plots of 340/380-nm ratio as a function of time for induced peripheral neurons that responded to IL-4 and capsaicin (e) and IL-31 and AITC (f). Black bars indicate time of application of each indicated stimulus.

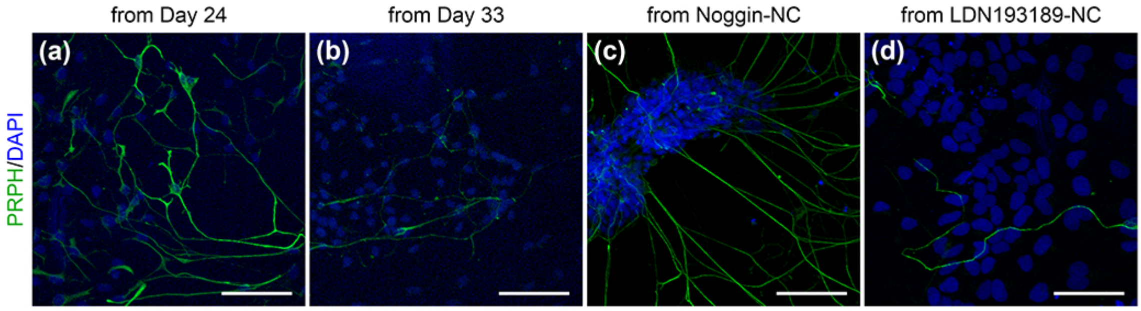

Supplementary Figure S5. Effect of culture conditions on the differentiation of peripheral sensory neurons

(a and b) Immunocytochemistry, showing the expression of PRPH (green) in differentiated cells from day 24 (a) (i.e. 14 days after NC induction on day 10) and day 33 (b). Peripheral neuronal differentiation was performed for 14 days from 201B7-derived NC cells.

(c and d) Immunocytochemistry showing the Noggin-induced differentiation of many (c) and few (d) peripheral neurons from 201B7-derived NC cells. Scale bars, 100 μm.

Supplementary Table 1.
Sequences of primer pairs used for quantitative real-time RT-PCR analyses.

Gene	Primer	Sequence (5'–3')
TFAP2A	Forward	AAGAGTTCACCGACCTGCTG
	Reverse	AGGGCCTCGGTGAGATAGTT
SOX10	Forward	GAGCTGGACCGCACACCTTGGG
	Reverse	AACGCCCACCTCCTCGGACCTC
NANOG	Forward	AGAGGTCTCGTATTTGCTGCAT
	Reverse	AAACACTCGGTGAAATCAGGGT
SOX9	Forward	TCAACGGCTCCAGCAAGAACAAG
	Reverse	ACTTGTAATCCGGGTGGTCCTTCT
SNAI1	Forward	CACTATGCCGCGCTCTTTC
	Reverse	GGTCGTAGGGCTGCTGGAA
SNAI2	Forward	TTTCTGGGCTGGCCAAACATAAGC
	Reverse	ACACAAGGTAATGTGTGGGTCCGA
PAX6	Forward	GCGGGTGACAAAATAGTTGTCTT
	Reverse	GCCAGGATGTCAAATCTCTCCA
BRN3A	Forward	GCACACATTCACACAGTGGTAACAG
	Reverse	AAGCTCAGCAGACATACATGGACAG
MRGX1	Forward	CTGGATTTCAAACTGGATTTGAGGA
	Reverse	ACCCTAGTCTGGTGACCCTGGA
GRP	Forward	CCGTGCTGACCAAGATGTACC
	Reverse	CTTCTATGAGACCCAGCAAATTCC
ISL1	Forward	TCTGTGGGCTGTTCACCAACTGTA
	Reverse	GCCGCAACCAACACATAGGGAAAT
TUBB3	Forward	ACAACGAGGCCTCTTCTCACAAGT
	Reverse	TAGTGACCCTTGGCCCAGTTGTTG
IL31RA	Forward	CGATTCAGGACAGTCAACAGTACCA
	Reverse	TTGACCGCACATCGCAGAG
IL4R	Forward	AAACACCCATGCCCAGCTGTA
	Reverse	GCCTTATGCCTGCTGTCTGGA
HRH1	Forward	CCAAGAGTGGTGGCAGCTCA
	Reverse	GCACGGGAACTCCATGTCAG
TRPV1	Forward	GAGTTTCAGGCAGACACTGGAA
	Reverse	CTATCTCGAGCACTTGCCTCTCT
TRPA1	Forward	CTGTGCAGGGCATGAATAATGAG
	Reverse	ATTTGTGGTGCACGCAATGA
RPS18	Forward	TTTGCGAGTACTCAACACCAACATC
	Reverse	GAGCATATCTTCGGCCCACAC
